# A soluble endoplasmic reticulum factor as regenerative therapy for Wolfram syndrome

**DOI:** 10.1038/s41374-020-0436-1

**Published:** 2020-05-04

**Authors:** Jana Mahadevan, Shuntaro Morikawa, Takuya Yagi, Damien Abreu, Simin Lu, Kohsuke Kanekura, Cris M. Brown, Fumihiko Urano

**Affiliations:** 1 Department of Medicine, Division of Endocrinology, Metabolism, and Lipid Research, Washington University School of Medicine, St. Louis, MO 63110 USA; 2 Department of Molecular Pathology, Tokyo Medical University, Tokyo, Japan; 3 Department of Pathology and Immunology, Washington University School of Medicine, St. Louis, MO 63110 USA

**Keywords:** Apoptosis, Type 2 diabetes

## Abstract

Endoplasmic reticulum (ER) stress-mediated cell death is an emerging target for human chronic disorders, including neurodegeneration and diabetes. However, there is currently no treatment for preventing ER stress-mediated cell death. Here, we show that mesencephalic astrocyte-derived neurotrophic factor (MANF), a neurotrophic factor secreted from ER stressed cells, prevents ER stress-mediated β cell death and enhances β cell proliferation in cell and mouse models of Wolfram syndrome, a prototype of ER disorders. Our results indicate that molecular pathways regulated by MANF are promising therapeutic targets for regenerative therapy of ER stress-related disorders, including diabetes, retinal degeneration, neurodegeneration, and Wolfram syndrome.

## Abstract (web summary)

Wolfram syndrome is a prototype of endoplasmic reticulum (ER) stress disorder characterized by diabetes, visual impairment, and neurodegeneration. Currently, there is no treatment that can halt or reverse the disease progression. Here, the authors show that mesencephalic astrocyte-derived neurotrophic factor-based regenerative gene therapy is an emerging therapeutic strategy for Wolfram syndrome and other ER stress-related disorders.

## Introduction

Growing evidence indicates that endoplasmic reticulum (ER) stress plays a critical role in β cell death in type 1 and type 2 diabetes, as well as in neurodegenerative disorders, including Parkinson’s disease and amyotrophic lateral sclerosis [1–5]. Despite the underlying importance of ER stress in β cell death, there is currently no diabetes treatment targeting the ER due to the complex nature of type 1 and type 2 diabetes. Our strategy for overcoming this challenge is to focus on a monogenic form of diabetes, Wolfram syndrome. Wolfram syndrome is a rare disease characterized by juvenile-onset diabetes mellitus, optic nerve atrophy, and neurodegeneration [6, 7]. As this syndrome is caused by mutations in the *WFS1* gene which is involved in ER calcium homeostasis and ER stress-mediated cell death, it is ideal for testing potential new treatments targeting the ER [8–14].

Mesencephalic astrocyte-derived neurotrophic factor (MANF) is a trophic factor whose expression and secretion is enhanced by ER stress and ER calcium depletion [15–18]. It has been demonstrated that MANF plays a critical role in the survival of ER stressed β cells and neurons [19, 20], raising the possibility that MANF-based treatment can be beneficial for patients suffering from ER stress-related disorders, including Wolfram syndrome. Here we show that MANF-based treatment prevents β cell death and enhances β cell proliferation in cell and mouse models of Wolfram syndrome. Our results indicate that molecular pathways regulated by MANF are promising drug targets for ER stress-related disorders, including β cell death in diabetes and Wolfram syndrome.

## Materials and methods

### Cell culture

*Manf* knockout INS-1 832/13 cells and *Wfs1* knockout INS-1 832/13 cells were created in collaboration with the Genome Engineering and Induced Pluripotent Stem Cell (iPSC) Center at Washington University using CRISPR-Cas9 genome editing techniques. INS-1 832/13 cells in which *Wfs1* expression can be suppressed by doxycycline-inducible shRNA directed against *Wfs1* (INS-1 DOX-sh*Wfs1*) were generated as described previously [9]. Briefly, INS-1 832/13 cells stably expressing pTetR were transduced with lentivirus expressing pTER(H1/tetO)-shWfs1. INS-1 DOX-sh*Wfs1* were cultured in 2 µg/ml doxycycline (MilliporeSigma, St. Louis, MO) for 48 h before isolation of protein and RNA, and glucose-stimulated insulin secretion (GSIS) assay. INS-1 832/13 cells stably overexpressing MANF (MANF-OE) were created by transducing INS-1 832/13 cells with lentivirus expressing human MANF. INS-1 832/13 cells were cultured in RPMI 1640 (Thermo Fisher Scientific, Waltham, MA) supplemented with 10% FBS (Thermo Fisher Scientific), 1 mM sodium pyruvate (Thermo Fisher Scientific), 100 nM β-mercaptoethanol (MilliporeSigma), and penicillin–streptomycin (Thermo Fisher Scientific). Tetracycline-free FBS (Takara Bio USA, Mountain View, CA) was used for culturing INS-1 DOX-sh*Wfs1*.

### Animal experiments

*Wfs1* β cell-specific knockout (β*Wfs1*^(−/−)^) mice were generated by breeding the Cre recombinase driven by rat insulin promoter (Rip2-Cre) transgenic mice (originally from Dr Pedro Herrera) with *Wfs1* floxed mice [21]. All animal experiments were performed according to procedures approved by the Institutional Animal Care and Use Committee at the Washington University School of Medicine (A-3381-01).

### Immunoblot analysis

INS-1 832/13 cells were washed in cold PBS and lysed with M-PER reagent (Thermo Fisher Scientific) containing Complete™ protease inhibitor cocktail (MilliporeSigma). The equivalent amounts of cell lysates were resolved by SDS-PAGE using 4–20% Mini-PROTEAN® TGX™ Precast Protein Gels (Bio-Rad Laboratories, Hercules, CA) and blotted onto Immobilon-P PVDF membrane (0.45 µm) (MilliporeSigma). The following primary antibodies were used for detecting the protein of interest; WFS1 antibody (Proteintech, Rosemont, IL), cleaved caspase-3, GAPDH, alpha-tubulin and beta-actin antibody (Cell Signaling Technology, Danvers, MA), and anti-MANF antibody (Abnova, Taipei City, Taiwan) at 1:1000 dilution. The secondary antibodies conjugated to horseradish peroxidase were obtained from Cell Signaling Technology. The detection was performed by enhanced chemiluminescence-select (GE Healthcare Bio-Sciences, Pittsburgh, PA). Fiji/ImageJ was used for the quantification of immunoblot.

### Quantitative PCR

Total RNA was extracted from INS-1 832/13 cells using the RNeasy Mini Kit (Qiagen, Germantown, MD) and reverse transcribed using High-Capacity cDNA Reverse Transcription Kits (Thermo Fisher Scientific). The expression of *Manf* and ER stress-related genes including Binding immunoglobulin protein (*Bip*), CCAAT/enhancer-binding protein-homologous protein (*Chop*), spliced X-box binding protein (Xbp1) (*sXbp1*), and Tribbles Pseudokinase 3 (*Trb3*) were detected by quantitative PCR (qPCR) using SYBR green reagents (Bio-Rad Laboratories). The qPCR was performed in triplicate for each sample. The primers sequences were: rat *Manf*, 5′-TGAGGTATCGAAGCCTCTGG-3′ and 5′-CTCGCAGATCTGGCTGTCTT-3′; rat *actin*, 5′-GCAAATGCTTCTAGGCGGAC-3′ and 5′-AAGAAAGGGTGTAAAACGCAGC-3′; rat *Bip*, 5′-TGGGTACATTTGATCTGACTGGA-3′ and 5′-CTCAAAGGTGACTTCAATCTGGG-3′; rat *Chop*, 5′-AGAGTGGTCAGTGCGCAGC-3′ and 5′-CTCATTCTCCTGCTCCTTCTCC-3′; rat *sXbp1*, 5′-CTGAGTCCGAATCAGGTGCAG-3′ and 5′-ATCCATGGGAAGATGTTCTGG-3′; rat *Trb3* 5′-ACCATGCGAGCCACATCTCTG-3′ and 5′-CTAGCCATACAGCCCCACCTC-3′.

### Primary islet culture

Mouse primary islets were taken from β*Wfs1*^(^^−/−^^)^ mice. The mice were anesthetized, and pancreata were infused with 5 ml of 0.45 mg/ml collagenase type V (MilliporeSigma) in Hank’s balanced salt solution without Ca^2+^ (Thermo Fisher Scientific). After surgical removal, pancreata were incubated for 12 min at 37 °C, and then hand-shaken for 2 min. Undigested acinar tissue was removed by using a 70-μm cell strainer and recovered tissues were washed twice with ice-cold Hanks’ balanced salt solution followed by centrifugation at 1100 rpm for 1 min. Islets were handpicked and preincubated in RPMI 1640 medium containing 10% FBS and antibiotics before experimentation. Islets of equal size were handpicked to generate 3–5 technical replicates for all experiments. Very large and very small islets were excluded. The results were obtained from at least three independent experiments.

### Human islet culture

Human islets were purchased from Prodo Laboratories (Aliso Viejo, CA), and cultured in CMRL-1066 medium (Corning Incorporated, Corning, NY) containing 5 mM glucose, 100 units/ml penicillin, 100 µg/ml streptomycin, 2 mM GlutaMAX (Thermo Fisher Scientific), 250 µg/ml gentamycin (Thermo Fisher Scientific), 10 mM HEPES (pH 7.4) (Thermo Fisher Scientific), and 10% FBS. Human islets (30 islets/well) were handpicked under a dissecting microscope.

### Insulin secretion assay

Primary mouse islets or INS-1 832/13 were cultured for 24 h and batches of ten islets were handpicked on the day of the experiment. Mouse islets or INS-1 832/13 were starved for 1 h in Krebs-Ringer bicarbonate-HEPES buffer (129 mM NaCl, 5 mM NaHCO_3_, 4.8 mM KCl, 1.2 mM KH_2_PO_4_, 1.2 mM MgSO_4_, 10 mM HEPES, and 1 mM CaCl_2_ at pH 7.4) containing 0.1% bovine serum albumin (KRBH/BSA). KRBH/BSA was supplemented with 2.8 mM glucose and then stimulated for 1 h at 37 °C in KRBH/BSA containing basal 5.5 mM or stimulatory 16.7 mM glucose. At the end of each incubation, supernatants were collected to measure insulin release, and cellular insulin contents were determined by acid–ethanol extraction followed by ELISA Rat/Mouse Insulin kit (MilliporeSigma).

### Cell proliferation

The islets isolated from humans donor or β*Wfs1*^*(*^^−/−^^)^ mice were dissociated by incubation with 0.25% trypsin-EDTA (Thermo Fisher Scientific) at 37 °C for 5 min and treated with MANF peptide (R&D Systems, Minneapolis, MN) 5 µg/ml for 5 days. Two-thirds of the medium were changed daily to fresh medium with MANF peptide. To monitor the cell proliferation rate, the BrdU cell proliferation assay kit (Cell Signaling Technology) was used following the manufacturer’s instruction.

### Caspase-3/7 activity in INS-1 832/13 cells

INS-1 832/13 cells were cultured in RPMI medium in a 96-well plate. Cells were treated with MANF peptide (5 µg/ml) for 24 h, and then exposed to thapsigargin (MilliporeSigma). Caspase-3 activity and cell viability were assessed using the Caspase-Glo® 3/7 assay kit and the CellTiter-Fluor™ cell viability Assay kit (Promega Corp., Madison, WI).

### Immunostaining

Pancreatic tissue sections were fixed, rehydrated and permeabilized with 0.1% Triton X-100 for 2 min. The sections were washed with 0.1% Tween-20 PBS (PBS-T) containing Image-It FX signal enhancer (Thermo Fisher Scientific) for 1 h and incubated with primary antibodies overnight at 4 °C [guinea pig anti-insulin antibody (1:100, Thermo Fisher Scientific), MANF (1:100, Abnova), and Ki67 (1:100, Cell Signaling Technology)]. The tissue sections were washed three times in PBS-T and incubated with secondary antibodies for 1 h at room temperature. Images were obtained with a Zeiss LSM 5 PASCAL confocal microscope with LSM Image software.

### Measurement of β-cell mass

For measurement of β-cell mass, every 40th pancreatic section was immunostained with guinea pig anti-insulin antibody (1:100, Thermo Fisher Scientific) and counterstained with hematoxylin. The β-cell mass for each mouse was quantified using Image Pro Plus software (Media Cybernetics, Rockville, MD) by obtaining the fraction of the cross-sectional area of pancreatic tissue (exocrine and endocrine) positive for insulin staining, and then multiplying this by the pancreatic weight.

### Measurement of apoptosis through TUNEL assay

Apoptotic cells were detected using the terminal deoxynucleotidyl transferase dUTP nick end labeling (TUNEL) method as per the manufacturer’s protocol (MilliporeSigma). For the determination of apoptosis, all β-cells per pancreatic sections (five sections per animal) were analyzed to count the total number of TUNEL-positive β-cells. An average of 150 islets was counted per animal and the percentage of TUNEL-positive cells was quantitated.

### In vivo administration of AAV vectors

The methods for AAV production are described in Supplemental Information. AAV was produced in collaboration with the Hope Center Viral Vectors Core at Washington University. Male (*n* = 3, 2–3 months of age) and female (*n* = 4, 3–4 months of age) β*Wfs1*^(−/−)^ mice received intraperitoneal injections of AAV9-CBA-IRES-GFP or AAV9-CBA-MANF-IRES-GFP at a final dose of 1 × 10^13^ viral genome particles diluted in saline per mouse. After 4 weeks of AAV administration, the pancreata were harvested. Dissected pancreas pieces were fixed in 4% formalin. Formalin-fixed paraffin-embedded sections were deparaffinized and rehydrated. To estimate the β-cell replication rate, pancreatic sections were immunostained with anti-insulin and anti-Ki-67 antibody, a marker for cellular proliferation. Overall, 1500–3000 β cells were counted in each animal.

### Data analysis

The values are expressed as mean ± SEM. All the statistical analysis was carried out with Prism 8 (ver 8.0.2). Comparisons among the group were done by Student’s *t* test. Multiple comparisons were performed by ANOVA followed by Tukey’s test. *P* < 0.05 was considered statistically significant.

## Results

### MANF confers protection against cell death induced by ER calcium depletion

**Fig. 1:**
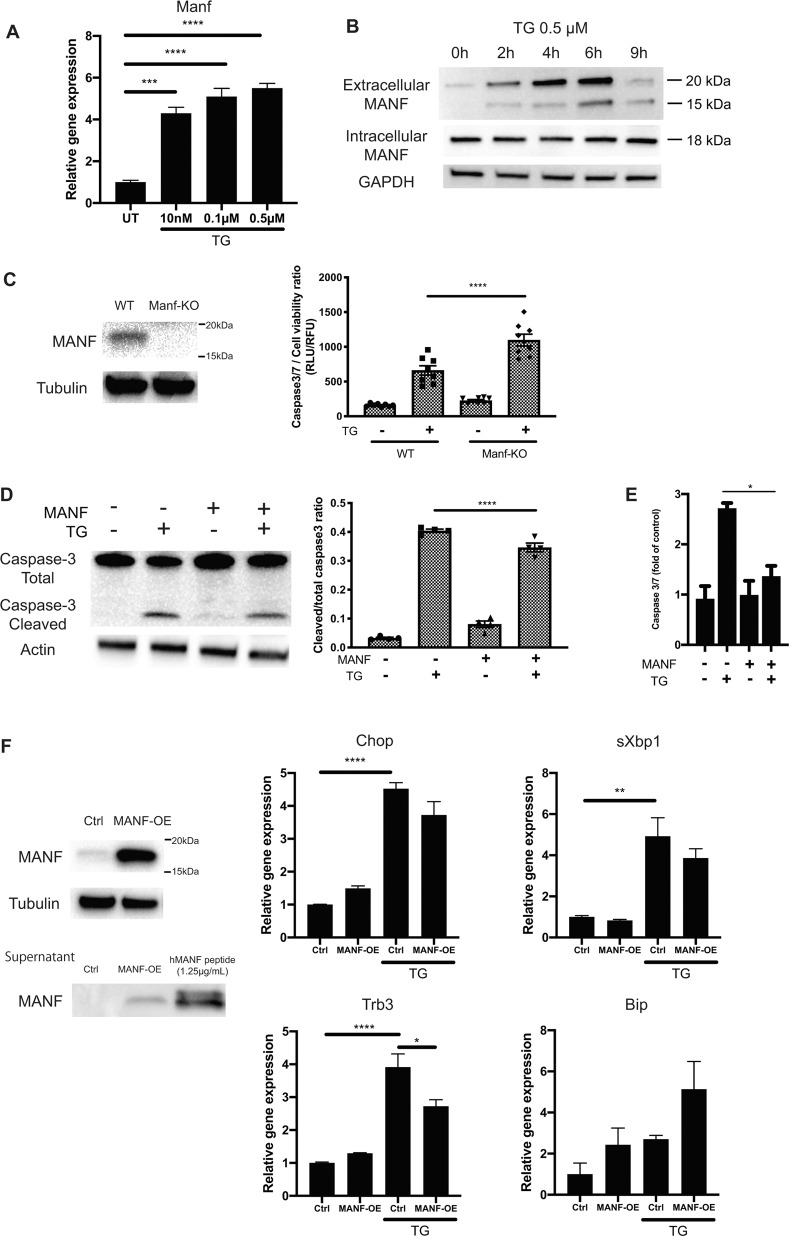
MANF expression and secretion are induced by ER calcium depletion leading to suppression of ER stress-mediated cell death. **a** qPCR analysis monitoring *Manf* mRNA expression levels in INS-1 832/13 cells treated with thapsigargin (TG) 10 nM for 24 h, 0.1 µM or 0.5 µM for 6 h. UT untreated (*n* = 3, ***P = 0.0001, ****P < 0.0001). **b** Western blot analysis monitoring extracellular and intracellular MANF levels. INS-1 832/13 cells were treated with 0.5 µM of TG for indicated times. **c** Left panel: western blot for evaluating the expression level of MANF protein in wild type (WT) and *Manf* knockout (Manf-KO) INS-1 832/13 cells. Right panel: caspase-3/7 activity normalized to cell viability in INS-1 832/13 cells treated with or without TG (0.1 µM for 4 h) (*n* = 8, ****P < 0.0001). **d** Western blot of cleaved caspase-3 in INS-1 832/13 cells pretreated with or without recombinant MANF peptide (5 µg/ml) for 24 h, and then challenged with TG (0.5 µM) for 6 h. Quantification of immunoblot analysis is shown in the right panel (*n* = 4, ****P < 0.0001). **e** The caspase-3/7 activity assay in INS-1 832/13 cells pretreated with recombinant MANF peptide (5 µg/ml) for 24 h, and then challenged with TG 0.5 µM for 6 h. **f** Left panel: western blot for evaluating the expression level of MANF protein in control (Ctrl) and MANF overexpressed INS-1 832/13 (MANF-OE) whole cell lysate and supernatant. Right panel: qPCR analysis monitoring the expression levels of *Chop, sXbp1*, *Trb3*, and *Bip* mRNA in Ctrl and MANF-OE INS-1 832/13 cells challenged with TG 0.5 µM for 6 h (*n* = 3, *P < 0.05, **P < 0.01, ****P < 0.0001).

We have recently shown that various β cell perturbants, including the loss of function of Wolfram syndrome 1 (*WFS1*) gene, induce ER calcium depletion and ER stress, leading to β cell death [10, 22]. It has been recently reported that loss of MANF in vivo leads to β cell death with ER stress elevation [20]. These considerations prompted us to monitor MANF expression levels in β cells under stressed conditions. Although *Manf* mRNA expression was not changed by *Wfs1* deficiency (Fig. S1), thapsigargin, which is a well-established ER calcium depletion inducer, increased *Manf* mRNA expression and MANF protein secretion in INS-1 832/13 cells (Fig. 1a, b). A smaller band of extracellular MANF corresponds to an isoform lacking RTDL domain which is prone to be secreted, and a larger band corresponds to an isoform containing RTDL domain which is glycosylated [15] (https://www.ncbi.nlm.nih.gov/protein/NP_001101653.1,XP_006243837.1). Intracellular fraction only contains an isoform with the C-terminal RTDL domain [23]. While *Manf* knockout INS-1 832/13 cells were more sensitive to ER stress-induced cell death (Fig. 1c), recombinant MANF peptide pretreatment reduced cell death in INS-1 832/13 cells treated with thapsigargin (Fig. 1d, e). Furthermore, mRNA expression level of tribbles pseudokinase 3 (*Trb3*), which is an ER stress-inducible gene, was significantly suppressed in INS-1 832/13 cells stably overexpressing MANF (MANF-OE) (Fig. 1f). *Trb3* is a proapoptotic component of ER stress signaling [24–26], suggesting that MANF might suppress the proapoptotic arm of ER stress signaling in those models.

### Effect of MANF on insulin secretion

**Fig. 2:**
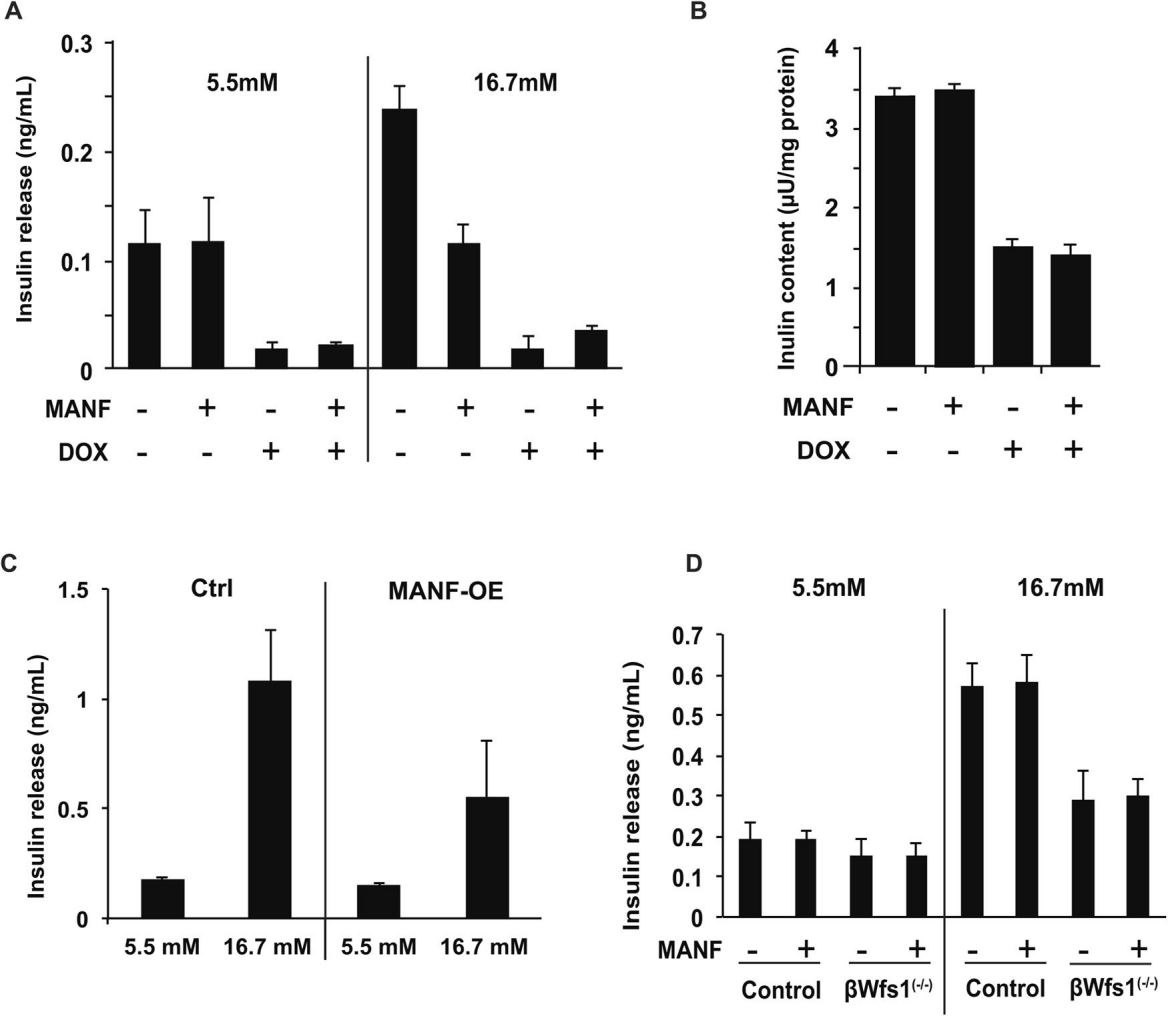
Effect of MANF on glucose-stimulated insulin secretion. **a** Doxycycline-inducible shRNA directed against *Wfs1* (INS-1 DOX-sh*Wfs1*) cells were treated with or without MANF peptide (5 µg/ml) for 24 h, and then treated with doxycycline (DOX). Insulin release was measured at basal (5.5 mM) glucose and stimulatory (16.7 mM) glucose conditions (*n* = 3, not significant). **b** Cellular insulin contents were measured after the 24 h pretreatment with MANF peptide (5 µg/ml) followed by DOX treatment (*n* = 3, not significant). **c** Glucose-stimulated insulin secretion on control (Ctrl) and MANF overexpressed INS-1 832/13 cells (MANF-OE). Insulin release was measured at 5.5 and 16.7 mM glucose conditions (*n* = 3, not significant). **d** Primary islets isolated from wild type (WT) and β cell-specific *Wfs1* knockout mice (β*Wfs1*^(−/−)^) were pretreated with MANF peptide (5 µg/ml) for 24 h. Insulin release was measured at 5.5 mM and 16.7 mM glucose (*n* = 3, not significant).

Since the loss of MANF in vivo can lead to β cell dysfunction, we studied the relationship between MANF and insulin secretion. We created INS-1 832/13 cells in which *Wfs1* expression can be suppressed by doxycycline-inducible shRNA directed against *Wfs1* (INS-1 DOX-sh*Wfs1*) [9]. Glucose-stimulated insulin secretion (GSIS) assays were performed in INS-1 DOX-sh*Wfs1* cells, MANF-OE INS-1 832/13, and primary mouse islets isolated from β cell-specific *Wfs1* knockout (β*Wfs1*^(−/−)^) mice treated with recombinant MANF peptide. As a consequence, MANF treatment or overexpression did not affect GSIS in those models (Fig. 2a–d).

### MANF activates proliferation of human primary islets

**Fig. 3:**
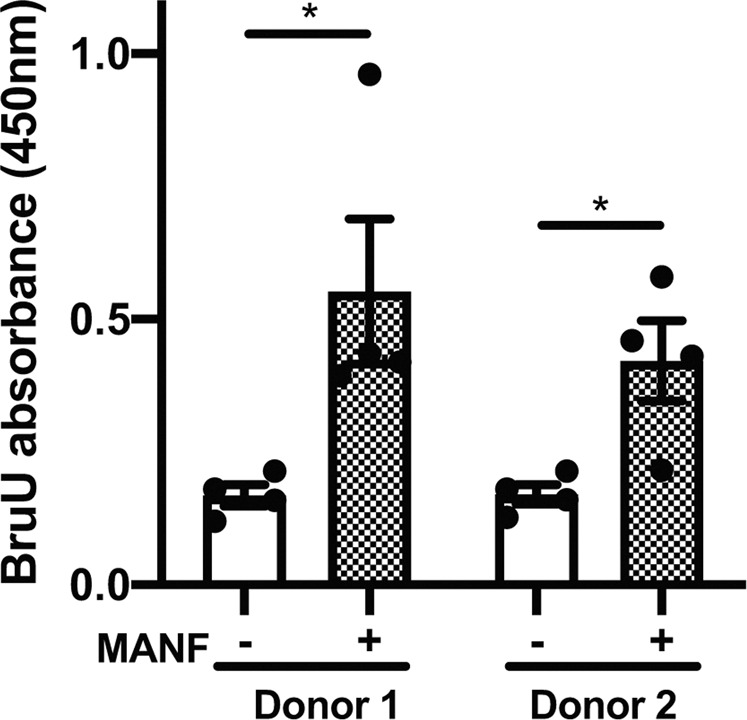
MANF activates proliferation of β cells. The BrdU assay monitoring the proliferation of human primary islets treated with or without MANF peptide (5 µg/ml) for 5 days (*n* = 4, *P < 0.05).

The fact that the suppression of ER stress can lead to β cell proliferation raised the possibility that MANF treatment might activate β cell proliferation [22, 23]. To test this idea, human primary islets were treated with recombinant MANF peptide and then their proliferation rates were assessed by the BrdU assay. Consequently, MANF treatment significantly induced the proliferation of human primary islets derived from two out of six donors (Fig. 3 and Supplementary Table).

### MANF-based treatment for Wolfram syndrome

**Fig. 4:**
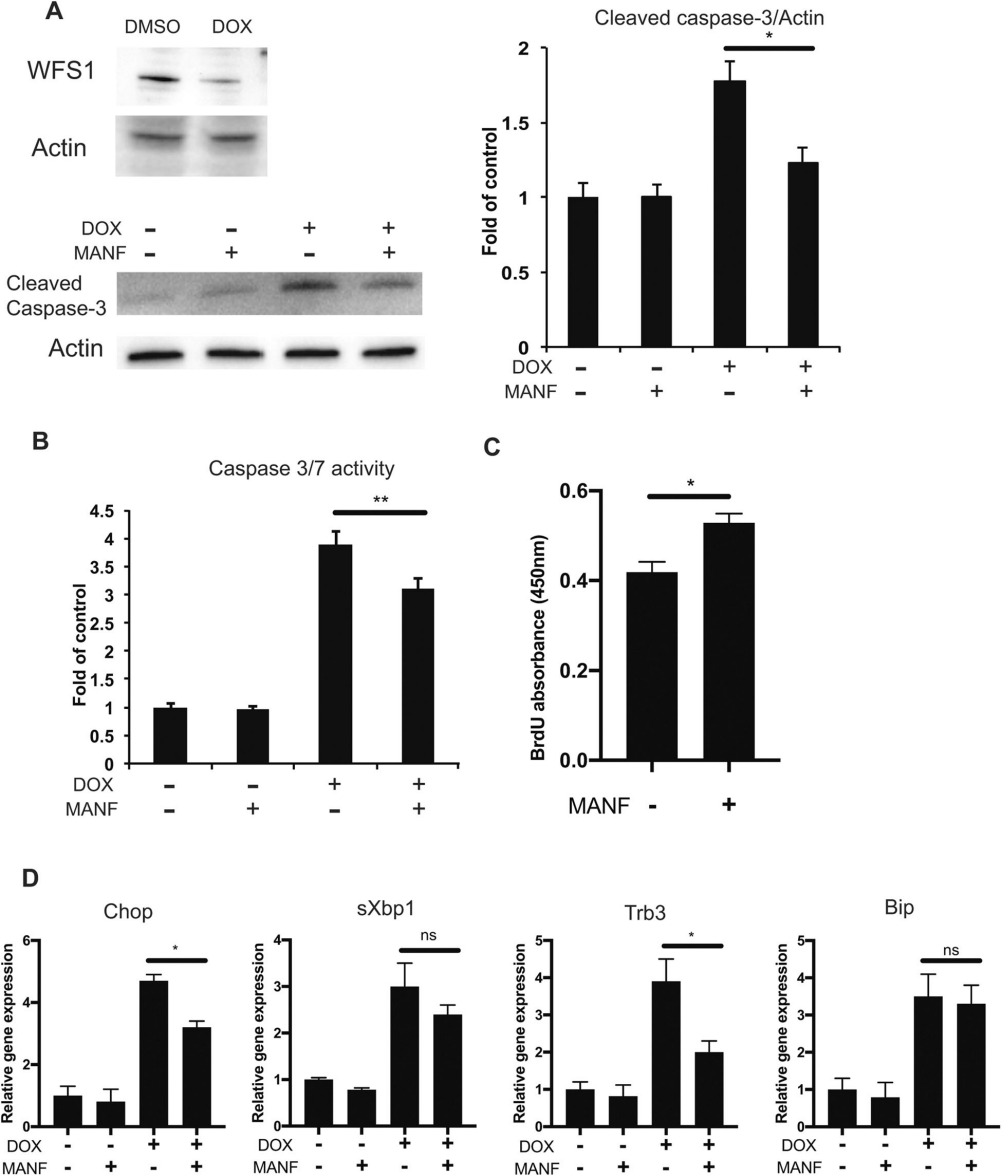
MANF attenuates cell death and activates cell proliferation in β cell models of Wolfram syndrome. **a** Immunoblot analysis of cleaved caspase-3 and actin in doxycycline-inducible shRNA directed against *Wfs1* (INS-1 DOX-sh*Wfs1*) cells. INS-1 DOX-sh*Wfs1* cells were untreated or pretreated with MANF peptide (5 μg/ml) for 24 h, and then treated with doxycycline (DOX) for *Wfs1* suppression. The quantified ratio of cleaved caspase-3 is shown in the right panel (*n* = 3, *P < 0.05). **b** Caspase-3/7 activity assay in INS-1 DOX-sh*Wfs1* cells. INS-1 DOX-sh*Wfs1* cells were untreated or pretreated with MANF peptide (5 μg/ml) for 24 h, and then treated with or without DOX for another 48 h (*n* = 3, **P < 0.01). **c** BrdU assay of β cell-specific *Wfs1* knockout mice (β*Wfs1*^*(*−/−)^) primary islets. The isolated islets were treated with or without MANF peptide (5 µg/ml) for 5 days (*n* = 4, *P < 0.05). **d** qPCR analysis monitoring the expression levels of *Chop*, *sXbp1*, *Trb3*, and *Bip* mRNA in INS-1 DOX-sh*Wfs1* cells. The cells were treated with or without MANF peptide (5 μg/ml) for 24 h, and then treated with DOX (*n* = 3, *P < 0.05).

We have previously shown that ER calcium depletion, followed by ER stress-mediated cell death, plays a role in the pathogenesis of Wolfram syndrome [10, 22, 27], which prompted us to consider the possibility that MANF-based treatment could prevent β cell death and activate β cell proliferation in Wolfram syndrome. Cell death induced by *Wfs1* knockdown in INS-1 DOX-sh*Wfs1* cells was prevented by recombinant MANF peptide treatment shown as cleaved caspase-3 protein and caspase-3/7 activity reduction (Fig. 4a, b). The proliferation of primary islets from β*Wfs1*^(−/−)^ mice, which is a mouse model of Wolfram syndrome [21], was also enhanced by MANF treatment (Fig. 4c). Moreover, MANF treatment suppressed the expression of proapoptotic ER stress markers (*Chop* and *Trb3*) in INS-1 DOX-sh*Wfs1* cells (Fig. 4d) and MANF overexpression improved the viability of *Wfs1* knockout INS-1 832/13 cells (Fig. S2).

**Fig. 5:**
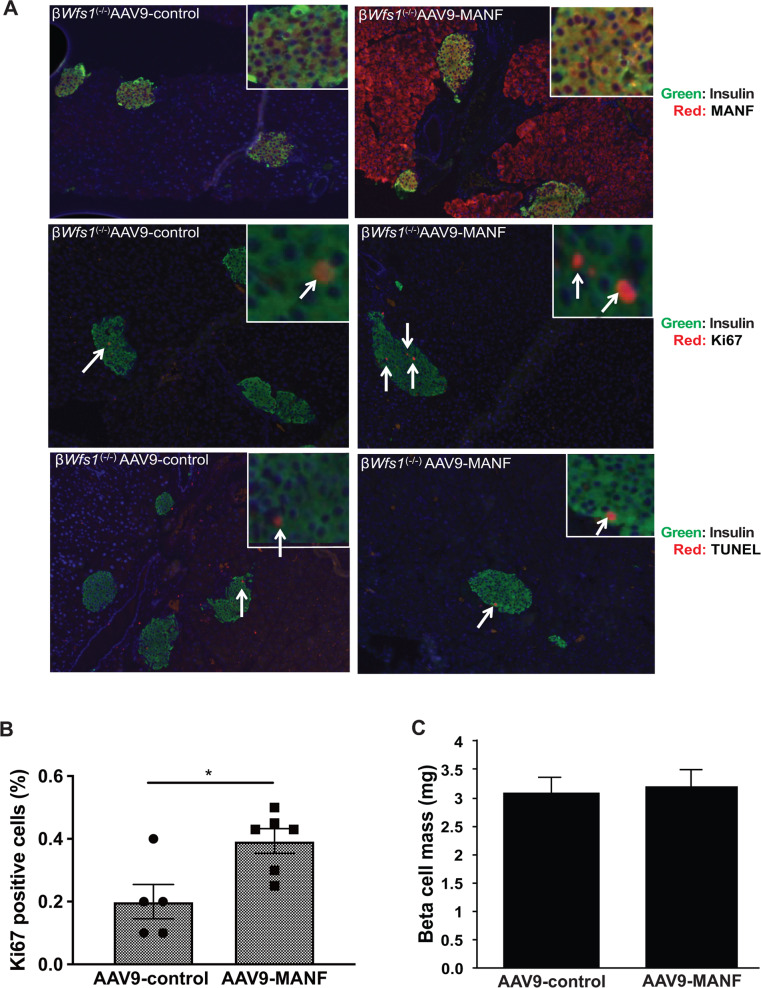
Overexpression of MANF by AAV9 enhances β cell proliferation in a mouse model of Wolfram syndrome. **a** Top panels: double immunofluorescence staining of insulin (green fluorescence) and MANF (red fluorescence) on pancreatic tissue sections from β cell-specific *Wfs1* knockout mice (β*Wfs1*^*(*−/−)^) taken 5 weeks after intraperitoneal injection of AAV-control (AAV9-CBA-IRES-GFP) or AAV9-MANF (AAV9-CBA-MANF-IRES-GFP) vector (*n* = 6 each group). Middle panels: double immunofluorescence staining of insulin (green fluorescence) and Ki67 (red fluorescence) of pancreatic sections from β*Wfs1*^*(*−/−)^ mice taken 5 weeks after intraperitoneal injection of AAV-control or AAV9-MANF vector (*n* = 6 in each group). Bottom panels: double immunofluorescence staining of insulin (green fluorescence) and TUNEL staining (red fluorescence) of pancreatic sections from β*Wfs1*^*(*−/−)^ mice taken 5 weeks after intraperitoneal injection of AAV-control or AAV9-MANF vector (*n* = 6 each group). The magnification is ×10 for each image, and ×40 for the right upper images. **b** Quantification of Ki67-positive β cells in AAV-control or AAV9-MANF injected β*Wfs1*^*(*−/−)^ mice (AAV-control, *n* = 5; AAV9-MANF, *n* = 6; *P < 0.05). **c** Quantification of β cell mass in AAV-control or AAV9-MANF injected β*Wfs1*^*(*−/−)^ mice (*n* = 6 in each group, not significant).

Next, we analyzed the effect of MANF on β cell proliferation in β*Wfs1*^(−/−)^ mice. Adeno-associated virus 9 expressing MANF (AAV9-MANF) was injected intraperitoneally into β*Wfs1*^(−/−)^ mice. We then monitored β cell proliferation for 5 weeks after the injections. Pancreas sections from those mice showed robust expression of MANF in islet β cells, as well as in exocrine pancreatic cells (Fig. 5a). We found that β cell proliferation rates in endocrine cells were higher in β*Wfs1*^(−/−)^ mice injected with AAV9-MANF than in those injected with control AAV9 (Fig. 5b). β cell mass was not increased in both groups (Fig. 5c). TUNEL-positive cells were rarely detectable in both groups (β*Wfs1*^(−/−)^ mice injected with AAV9-control, 0.09 ± 0.02%; β*Wfs1*^(−/−)^ mice injected with AAV9-MANF, 0.08 ± 0.02%) (Fig. 5a). Although MANF was also overexpressed in exocrine pancreatic cells, proliferation rates of these cells were comparable with control cells, suggesting that the proliferative effect of MANF might be specific for pancreatic β cells (Fig. S3). Collectively, these results indicate that MANF enhances β cell survival and proliferation in cell and mouse models of Wolfram syndrome.

## Discussion

Wolfram syndrome is characterized by juvenile-onset diabetes, optic nerve atrophy and, neurodegeneration due to ER stress-mediated cell death [6, 28], and has been established as a prototype of ER stress disease [8, 9, 11–14, 21, 29]. Since there is no treatment that can stop or even slow the progression of this syndrome currently, developing the novel treatment has been an urgent task.

Increasing evidence indicates that MANF possesses regenerative and cytoprotective effects. In the mouse pancreas, MANF overexpression was found to induce the proliferation of pancreatic β cells [20]. Systematic MANF overexpression or recombinant MANF peptide delivery protects the liver of old mice from inflammation and hepatocyte apoptosis [30]. Notably, recombinant human MANF peptide protects human β cells from cytokine-induced ER stress and cell death, and induces β cells proliferation [31]. In this study, we show that MANF treatment activates the proliferation of β cells in human islets and prevents ER stress-mediated β cell death and enhances β cell proliferation in cell and mouse models of Wolfram syndrome. These results broaden the possibility of developing the new treatments for Wolfram syndrome using adeno-associated virus expressing MANF or recombinant MANF peptide. To elucidate the efficacy of MANF treatment, further experiments using the other Wolfram syndrome model mice, or β cells which are differentiated from Wolfram syndrome patient-derived iPSCs would be required [12]. On the other hand, MANF treatment did not change insulin secretion and insulin content in INS-1 832/13 cells. These results are in line with the previous report using EndoC-βH1 cells [31]. Moreover, even though MANF overexpression activated the β*Wfs1*^*(*−/−*)*^ mice β cell proliferation, the β cell mass of these mice was not changed. A longer overexpression might be needed to study the effect of MANF on the β cell mass.

MANF was originally isolated from astrocytes as a novel neurotrophic factor [15]. It has been reported that MANF regulates the NF-kB signaling pathway, which is considered to be activated through their receptors [31, 32]. However, receptors for MANF have not been identified. Further studies are required to identify these receptors and their signaling pathway in order to develop treatments based on small molecules that act as MANF receptor agonists.

Our results are also relevant to other diseases related to ER stress. Genetic, clinical, and experimental evidence indicates that ER stress-mediated cell death is an important pathogenic component in human chronic disorders, including type 1 and type 2 diabetes, retinal degeneration, Parkinson’s disease, amyotrophic lateral sclerosis, inflammatory bowel disease, and multiple sclerosis [3, 33–39]. It has been reported that plasma MANF protein levels decline with age in flies, mice, and human [30]. In contrast, circulating MANF levels are known to increase in children with type 1 diabetes as compared with control subjects [40]. ER stress in β cells has been linked to autoimmunity and cytokine-mediated β cell death during the onset and progression of type 1 diabetes [41–47]. Thus, increased MANF levels in patients with type 1 diabetes may be an adaptive response to ER stress in β cells. MANF mutations have been reported in a patient with type 2 diabetes [48]. In such disorders, MANF-based therapy may suppress ER stress-mediated cell death and delay the progression of the disease.

Collectively, our results provide a rationale for identifying signaling molecules regulated by MANF, including its receptor, so that we may develop novel regenerative therapy for ER stress-related disorders, including diabetes, retinal degeneration, and Wolfram syndrome.

## Supplementary information

Supplementary information
