# Supplementary material for: A soluble endoplasmic reticulum factor as regenerative therapy for Wolfram syndrome
Source: Lab Invest. 2020 May 4;100(9):1197–207. doi: 10.1038/s41374-020-0436-1 (PMC7438202; doi:10.1038/s41374-020-0436-1)
Supplement: Supplementary file 1 — Supplementary information [file 41374_2020_436_MOESM1_ESM.pdf]

Supplemental Information

AAV vector production

The packaging cell line, HEK293, was maintained in Dulbecco's modified Eagles medium (DMEM), supplemented with 5% fetal bovine serum (FBS), 100 units/ml penicillin, 100 μg/ml streptomycin in a 37°C incubator with 5% CO_2. The cells were plated at 30-40% confluence in CellSTACS (Corning Incorporated, Corning, NY) 24 h before transfection (70-80% confluence when transfected). 730 μg of pAAV 2/9, 1180 μg pHelper, and 590 μg rAAV transfer plasmid containing the gene of interest were co-transfected into HEK293 cells using the calcium phosphate precipitation. The cells were incubated at 37°C for 3 days before harvesting. The cells were lysed by three freeze/thaw cycles. The cell lysate was treated with 25U/ml of Benzonaze at 37ºC for 30 min. After centrifugation at 10,000 g for 20 min, the supernatant was collected and precipitated in PEG (final concentration of PEG: 8%) overnight. After centrifugation at 2,600 g for 30 min, the supernatant was discarded and the pellet was re-suspended with lysis buffer (150 mM NaCl, 50 mM Tris-HCl, pH 8.5). In a 38.5 ml polyallomer tube, 5 ml of CsCl (1.5 g/ml) was added and 12 ml of CsCl (1.37 g/ml) was overlaid along the side of the tube. The sample was ultracentrifuged at 24,000 rpm (182,000 g), 20ºC for 24 h. A 21-gauge needle was inserted through the bottom side of the centrifuge tube. A 1-ml fraction was collected. Vector-containing fractions were pooled and diluted with 1.37 g/ml CsCl. The sample is loaded to a 13.5 ml Quick-seal tube and centrifuged with an ultracentrifuge at 67,000 rpm (384,000g), 20ºC for 16-20 h. 0.5-ml fractions were collected. Vector-containing fractions were pooled and then concentrated with Vivaspin 20 100K concentrator (Sartorius, Göttingen, Germany). The vector titer was determined by Dot blot assay. All of the procedures above were performed by Hope Center Viral Vectors Core at Washington University in St. Louis.

Supplemental Figure

Figure S1

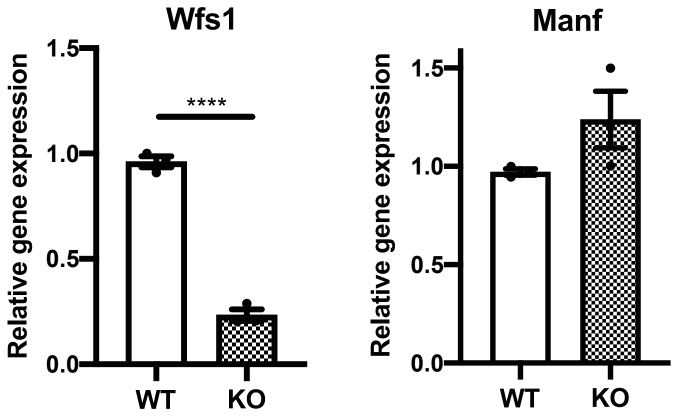

Figure S1. ***Manf*** **expression level is not significantly changed by** ***Wfs1*** **deficiency in INS-1 832/13 cells.**

qPCR analysis of *Wfs1* and *Manf* in *Wfs1* wild type (WT) and knockout (KO) INS-1 832/13 cells. Total RNA was extracted from INS-1 832/13 cells as described in the Method section of the main text. *Manf* expression level was not significantly elevated in *Wfs1* KO INS-1 832/13 cells. Also, *Manf* expression was not changed even in *Wfs1* knockdown INS-1 832/13 cells (data not shown). (n=3, **** $P<0.0001$)

Figure S2

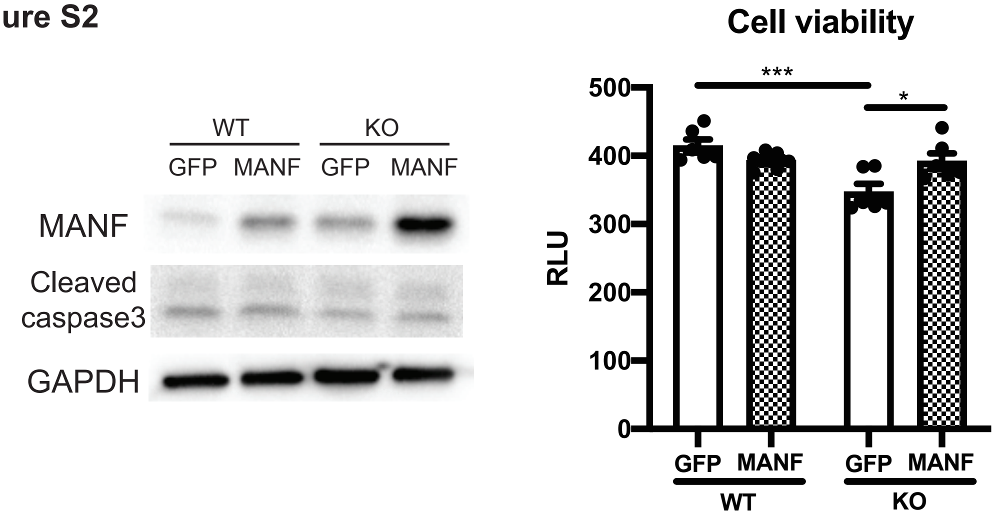

Figure S2. Viability of *Wfs1* knockout INS-1 832/13 cells was increased by MANF overexpression. GFP or human MANF were overexpressed in *Wfs1* wild type (WT) and knockout (KO) INS-1 832/13 cells by lentivirus transduction at multiplicities of infection (MOI) 5. Twenty four hours after the virus transduction, the medium was changed to fresh medium not containing lentivirus. After another 24 h, total protein was collected for western blot (*left panel*). Cell viability assay (*right panel*) was performed as described in the Method section of the main text. MANF overexpression increased the cell viability of *Wfs1* knockout INS-1 832/13 cells. (n=6, * $P<0.05$, *** $P<0.001$)

Figure S3

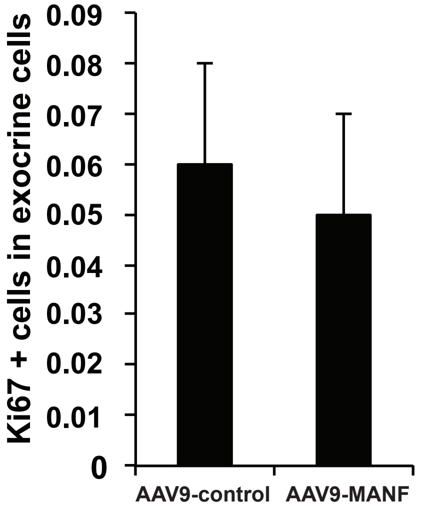

Figure S3. The proliferative effect of MANF for exocrine pancreatic cells

Quantification of Ki67 positive exocrine pancreatic cells in AAV9-control or AAV9-MANF injected β*Wfs1*$^{(-/-)}$ mice (n=6 in each group, not significant).

Supplemental Table

Gender	Age	BMI	HbA1C (%)	History	Beta cell proliferation by MANF peptide
Female	51	22.6	5.6	Ruptured cerebral artery aneurism leading to brain death	Yes
Male	31	24	5.3	Military person died of self-inflicted gun shot wound to head	Yes
Male	42	32.8	5.6	Brain dead from stroke with intra-cranial hemorrhage	No
Male	68	35.6	6.5	Type-2 diabetes, brain dead from stroke	No
Female	23	24.5	4.9	Brain dead following blunt trauma from motor vehicle accident	No
Male	23	21.4	5.5	Died from motor vehicle accident with head trauma	No
